# The complete chloroplast genome sequence of the threatened trident maple *Acer buergerianum* (Aceraceae)

**DOI:** 10.1080/23802359.2017.1325345

**Published:** 2017-05-12

**Authors:** Jia-Huan Xu, Hai-Bo Wu, Li-Zhi Gao

**Affiliations:** a Faculty of Life Science and Technology, Kunming University of Science and Technology, Kunming, China;; b Plant Germplasm and Genomics Center, Germplasm Bank of Wild Species in Southwest China, Kunming Institute of Botany, The Chinese Academy of Sciences, Kunming, China

**Keywords:** Chloroplast genome, *Acer buergerianum*, phylogenomic analyses

## Abstract

Trident maple *Acer buergerianum* Miq., belonging to the family of Aceraceae, is an important ornamental tree. Here, we report the complete chloroplast genome sequence of *A. buergerianum*. The circular genome was 156,477 bp in size, and comprised a pair of inverted repeat (IR) regions of 26,090 bp, a large single-copy (LSC) region of 86,246 bp and a small single-copy (SSC) region of 18,062 bp. It contained 134 genes, including 89 protein-coding genes, 40 transfer RNA genes, and 8 ribosomal genes. Maximum likelihood phylogenomic analysis shows that *A. buergerianum* is closely related to other *Acer* species, including *A. miaotaiense*, *A. morrisonense*, and *A. davidii*.

Trident maple *Acer buergerianum* Miq., a member of the family of Aceraceae, is native to eastern China (Hayashi 2007) and Japan (Matsue & Takeda 2009). As an important ornamental tree, it has enormous commerce value. In recent decades human activities have seriously destroyed natural habitats, leading to the loss and quick decline of a large number of natural populations of wild trident maple in China. As plant chloroplast sequences can provide helpful information to reconstruct complex evolutionary relationships, they have been widely applied for species identification and phylogenetic analysis during the past years (Jansen et al. 2007; Moore et al. 2007; Parks et al. 2009; Ruhsam et al. 2015). The information of chloroplast genomes has been extensively applied in understanding plant genetic diversity and conservation genetics (Ye et al. 2014; Zhang et al. 2016). Thus, the obtainability of chloroplast genome of *A. buergerianum* will prominently advance the conservation efforts of this threatened plant species that harbours valuable gene sources for the breeding program of trident maple.

In this study, we sequenced, assembled, and annotated the chloroplast genome of *A. buergerianum* using high-throughput genome sequencing technology. Young, fresh and healthy leaves of *A. buergerianum* were collected from a wild trident maple tree from Xupu, Hunan Province, China (110.6086E, 27.9101N). Total genomic DNA was extracted using a modified CTAB method (Doyle & Doyle 1987). According to the Illumina’s protocol, the pair-end libraries were constructed and then sequenced using Hiseq2000 platform, obtaining ∼50.1 million high-quality clean reads. With *A. miaotaiense* (Zhang et al. 2016) as a reference, we assembled and annotated the cp genome using CLC Genomic Workbench v7.5 (CLC BIO, Denmark) and Dogma (https://dogma.ccbb.utexas.edu/), respectively. The annotated genomic sequence was deposited into the GenBank database under the accession number of KY419137. The complete chloroplast genome is 156,477 bp in size, containing a large single copy region (LSC) of 86,246 bp, a small single copy region (SSC) of 18,062bp, and a pair of 26,090 bp inverted repeat regions (IRs). The chloroplast genome harbours 134 genes, including 89 protein-coding genes, 40 tRNA genes and 8 rRNA genes. The base composition is asymmetric (A: 30.65%; C: 19.30%; G: 18.58%; T: 31.47) with an overall GC content of 37.88%.

To determine the phylogenetic position of *A. buergerianum*, we constructed the phylogenetic tree between *A. buergerianum* and 10 other plant species based on whole chloroplast genome sequences with maximum likelihood (ML) method using MEGA7 (Kumar et al. 2016) (Figure 1). As expected, *A. buergerianum* is closely related to other *Acer* species, including *A. miaotaiense*, *A. morrisonense*, and *A. davidii*, and they form a clade together with *Dipteronia sinensis* belonging to the family Aceraceae with a strong bootstrap value of 100%. The complete chloroplast genome of *A. buergerianum* will provide valuable genetic data for the conservation management of the precious ornamental tree species.

**Figure 1.**
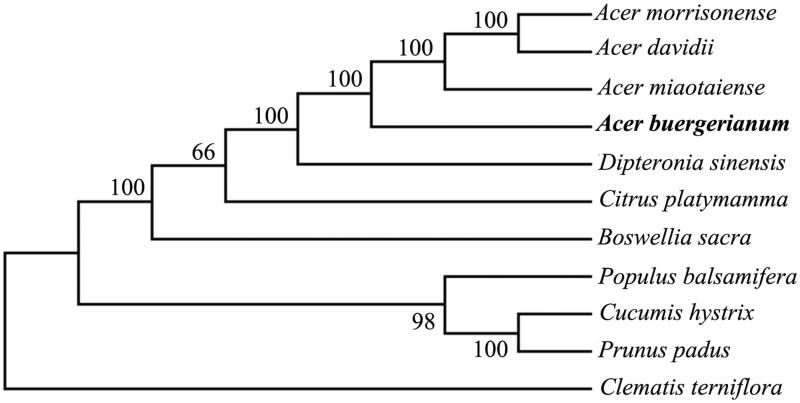
Maximum likelihood phylogenetic tree of *A. buergerianum* with 10 other plant species based on complete chloroplast genome sequences using *Clematis terniflora* as outgroup. Numbers in the nodes are bootstrap values from 1000 replicates. Accession numbers are listed as below: *Acer morrisonense* NC_029371.1, *Acer davidii* NC_030331.1, *Acer miaotaiense* NC_030343.1, *Dipteronia sinensis* NC_029338.1, *Citrus platymamma* NC_030194.1, *Boswellia sacra* NC_029420.1, *Populus balsamifera* NC_024735.1, *Cucumis hystrix* NC_023544.1, *Prunus padus* NC_026982.1, *Clematis terniflora* NC 028000.1.
